# COVID-19 related perception among some community members and frontline healthcare providers for NTD control in Ghana

**DOI:** 10.1186/s12879-022-07084-0

**Published:** 2022-01-30

**Authors:** Collins S. Ahorlu, Daniel Okyere, Sellasie Pi-Bansa, Joseph Otchere, Benjamin Marfo, Kofi Asemanyi-Mensah, Joseph L. Opare, Elizabeth F. Long, Dziedzom K. de Souza

**Affiliations:** 1 Epidemiology Department, Noguchi Memorial Institute for Medical Research, College of Health Sciences, University of Ghana, Legon, Accra, Ghana; 2 Parasitology Department, Noguchi Memorial Institute for Medical Research, College of Health Sciences, University of Ghana, Legon, Accra, Ghana; 3 Neglected Tropical Diseases Programme, Ghana Health Service, Accra, Ghana; 4 Neglected Tropical Diseases Support Center, Task Force for Global Health, Decatur, GA USA

## Abstract

**Introduction:**

The Coronavirus Disease 2019 (COVID-19) pandemic has resulted in a major breakdown of health service provision in the fight against neglected tropical diseases (NTDs). COVID-19 may impact NTDs service delivery in varied ways. As the Ghana NTD programme planned to resume MDA activities, we examined the COVID-19 related perceptions and practices among some community members and frontline health workers for NTD control activities in the country.

**Methods:**

The study was conducted in seven communities in the Ahanta West district of Ghana. This was a qualitative study using in-depth interviews (IDI) and focus group discussions (FGDs) for data collection. Participants were purposively selected from varied backgrounds to represent both beneficiaries and service providers directly involved in NTD programme implementation. Trained and experienced qualitative data collectors conducted the FGD and IDI sessions in the local Twi language, while health worker sessions were conducted in English. Discussions were audio-recorded and transcribed directly into English. Data was analysed using an iterative process. The transcripts were pre-coded using the broad themes, entered into a computer using Microsoft Word, and then imported into the MAXQDA software for thematic content analysis to select relevant representative narratives for presentation.

**Results:**

Participants were aware of the COVID-19 pandemic and referred to it appropriately as ‘coronavirus’, COVID-19, and often as ‘the new disease’. Though many respondents could not describe the route of transmission, most of them reported that it is transmitted through touch or sharing of common items. They reported some signs/symptoms like fever, headache and difficulty breathing, and prevention methods like the use of hand sanitiser, washing of hands and sneezing appropriately. Respondents have reported that COVID-19 has negatively affected their daily lives by limiting their movements and therefore work. It also came to light that COVID-19 has also negatively affected the NTD programme implementation, especially mass drug administration (MDA) activities, leading to the postponement of the yearly MDA. The COVID-19 pandemic has negatively affected clinic attendance; people are afraid that they may be tested for COVID-19 at the clinic.

**Conclusion:**

COVID-19 has negatively affected the NTD programme. Education and the provision of personal protective equipment will be required to build the confidence of frontline care providers including community drug distributors and community members in order to enhance quality service and participation in future MDA activities.

**Supplementary Information:**

The online version contains supplementary material available at 10.1186/s12879-022-07084-0.

## Introduction

Neglected Tropical Diseases (NTDs) are communicable diseases that affect the bottom 1 billion people who are also the most marginalised in society [1]. Lymphatic filariasis (LF) is one of the neglected tropical diseases (NTDs) targeted for elimination by the World Health Organization (WHO) as a public health problem. In the year 2000, the Global Programme to Eliminate Lymphatic Filariasis (GPELF) was set up with the aim to eliminate the disease by the year 2020 by implementing two main strategies: (i) interrupting the transmission of the disease through yearly treatment of the entire endemic communities [2, 3] and (ii) alleviating the suffering associated with LF morbidity and disability [4]. The drugs of choice for the MDA were either the combination therapy of albendazole together with ivermectin and or diethylcarbamazine, depending on the geographic setting and co-endemicity with diseases such as onchocerciasis and loa loa [5].

Ghana is endemic for several neglected tropical diseases; however, the focus of this paper is on LF, which is endemic in 98/216 districts. Since 2001 the country has implemented the GPELF strategy, with significant progress made in the control of the disease [6]. There are challenges to the elimination programme, which is evident in the fact that, despite several years of MDA, transmission persists in 17 of 98 endemic districts [7], a phenomenon that has given such districts the title of “hotspots”. It thus, appears that the country is not able to attain optimum treatment coverage [8] and this situation is likely to be imparted by the COVID-19 pandemic, which has stalled MDA activity in 2020.

The Coronavirus Disease 2019 (COVID-19) pandemic has resulted in a significant breakdown of health service provision in the fight against NTDs [9]. The World Health Organization (WHO) recently launched a new roadmap targeting the control and elimination of NTDs by 2030 [10]. The potential impact of COVID-19 on delaying the attainment of the 2030 control and elimination goals include shortfall in funding and political commitment, challenges with NTD service delivery as a result of the diversion of human capital, and impact of social mobilisation and community acceptance of interventions post-COVID-19 [11]. Other potential impacts include the resurgence of infections [12] and supply chain disruptions for essential diagnostics [13].

In April 2020, the WHO issued an interim guidance on the conduct of NTD activities, effectively suspending all NTD activities, except those requiring ongoing patient management [14]. In Ghana, the government banned travel, gatherings, and school activities on 15th March 2020, followed by a partial lockdown in Accra, Kumasi, and Kasoa, the main hotspots fuelling transmission. However, the government started to ease restrictions on 31st May 2020. While the Ghana NTD programme planned to resume mass drug administration (MDA) activities, we looked at the perception of some community members and health workers at the forefront of NTD control activities in Ghana. The aim is to understand the effect of COVID-19 on NTD interventions, especially the annual mass drug administration (MDA) for lymphatic filariasis (LF) elimination, giving that MDA was not implemented in 2020 due the pandemic. The question then is, how will the pandemic related restrictions and the availability and use of PPE affect MDA implementation and participation, when it resumes? Understanding these issues may help the program to take steps to ensure a successful resumption of MDA activities in this hotspot district.

## Methods

### Study sites

Data were collected in September 2020 in the Ahanta West district of Ghana, an area with persistent transmission of lymphatic filariasis (LF) [15]. The district is inhabited mainly by the Ahanta speaking people belonging to Ghana’s larger Twi speaking group. Farming and fishing are the main occupations of the people. However, small scale mining and trading also employeds many residents. Ghana’s budding oil industry is partly located off the shores of the district. The Ahanta West district started MDA implementation in 2002 and therefore has received more than 16 rounds of MDA without interruption of transmission [15]. For this study, a community was randomly selected from each of the seven subdistricts forming the district. All communities with a health facility in each subdistrict were numbered, put in a bowl, and picked to represent the subdistrict. Selecting a community in each subdistrict ensured that the selected communities were spread across the entire district. The subdistricts are the local health administration units within the Ghana Health Service structure in a district, whose demarcation is usually based on promoting the geographical accessibility of health services within a district.

### Study design

This qualitative study used in-depth interviews (IDI) and focus group discussions (FGDs) for data collection. This study design was chosen for this study because of the need to collect quick and probing information from frontline healthcare workers and community members for quick decision making to inform the resumption of MDA activities.

### Community entry

Prior to the initiation of data collection, the study team informed regional, district, and health facility officials of the Ghana Health Service (GHS) about the study objectives, methods, and timeline. Upon arrival in each community, the study team met the officials of the subdistrict health facilities, briefed them about the study and what is expected of them. The health center officials then facilitated meetings with local leaders and locally elected assembly members. After gaining the support of community leaders, the team, assisted by community-based volunteers and an officer of the district health management team, recruited study participants (Additional file 1).

### Data collection

All participants and data collectors were dressed in appropriate Personal Protective Equipment (PPE) and kept acceptable physical distancing. Trained and experienced qualitative data collectors conducted the FGDs and IDI sessions in the local Twi language; health workers were interviewed in English. A moderator, note taker, and supervisor facilitated each FGD. The moderator followed a semi-structured discussion guide, developed around key themes of interest. Each FGD session lasted approximately 60 to 90 min while the in-depth interview sessions lasted 30–45 min. The discussions were audio recorded and transcribed directly into English on an on-going basis (the recorded audio was transcribed directly into English because it was established during pre-testing that transcribing directly into English was faster and convey the best meaning of what was said in the local Twi language than first transcribing into the local Twi language before translating it into English language). Following each day’s work, the study teams debriefed to identify emerging themes that required further probing and to ensure data quality from the field. A supervisor routinely reviewed all English translations and provided continuous feedback to the data collection team.

Participants were selected variedly as follows: There were only two district level disease control officers, and both were purposively selected to participate in the study. Also, the CHNs were purposively selected from each of the six subdistrict level health facilities with the support of the facility-in-charge, taking into consideration their prior involvements in MDA activities. The CDDs were selected from the list of CDDs made available by the district health directorate using a simple random sampling technique. Using a simple random sampling technique, community members, aged 18–60 years, were selected from community-based registers of the six communities. The FGD participants were selected based on age and sex, these variables are considered to influence free and natural discussion in rural Ghana [16], hence the need to group participants into three age groups to reduce the age gaps among participant in each session. This promotes effective interactions among participants with very little or no age limitations. To follow the national COVID-19 protocols, we limited FGD participants to six per session. The FGDs allowed us to gain divergent viewpoints as well as consensus positions among participants while the IDI allowed individuals, space to share their experiences and perceptions on COVID-19 in the context of MDA activities in the district. Information solicited included: knowledge on the COVID-19 pandemic, its transmission, symptoms, impact on NTD programmes, and attendance to health facilities. Other topics included the willingness to participate in NTD programmes (mainly mass drug administrations) during the COVID-19 pandemic, other health delivery services, community members’ perception on PPE use, and whether the NTD programme should continue with its activities during and post COVID-19.

### Data analysis

Data were analysed using an iterative process, beginning with the first interview, and continuing throughout data collection via daily debriefings where data and emergent themes were reviewed. The dialogues were transcribed directly into English and entered into a computer using Microsoft Word, which was then imported into MAXQDA software for qualitative data analysis. The thematic categories were developed based on the issues for the study and were operationalized by the question guides used for data collection. During analysis, we developed themes as attributes, descriptors, or concepts that are explicit for organizing a group of related ideas that enable us to answer the study questions. Then, related issues that have a common point of reference were coded to generally unify ideas regarding the subject of our investigation [17–21]. The coded segments or narratives were categorised for deductive content analysis to select a representative and relevant responses that represented majority and minority views for presentation. Coding was carried out using predetermined themes based on the research questions and key areas of interest [20]. A codebook was developed and refined by the first author as the analysis progressed. All transcripts were coded by the second author and reviewed by the first and last authors.

The reliability of the qualitative data collected was ensured by first pretesting the tools among similar populations. This also help to ensure that the tools were understood consistently across respondents. Findings from the pretesting was used to refine the tool before commencement of final data collection. Secondly, the recorded audios were transcribed by the same person, but four audios—2 each of FGDs and IDIs were unanimously transcribed by a second person to ensure consistency and reliability of the data. Two IDI transcripts were shared with two respondents to confirm whether their views were accurately captured, they were both happy with the scripts and confirmed that their views were adequately captured. Triangulation of the data sources was done to ensure that data from various sources complement each other, however, divergent views or positions are represented in the result. Finally, verbatim quotations from respondents’ narratives were presented in the results as much as possible.

### Ethical approval

The study was reviewed and approved by the Noguchi Memorial Institute for Medical Research (NMIMR IRB CPN 021/19-20). All participants provided written informed consent prior to participation. Participants’ participation was voluntary, and respondents reserved the right to withdraw from the study at any time. Participants were informed about the research objectives and were ensured of confidentiality of information provided.

## Results

### Socio-demographic characteristics of study participants

In all, 91 individuals participated in the study, comprising 19 in-depth interviews and 12 focus group discussions as presented in Table 1. In all, there were 72 participants in the FGD sessions with equal numbers of males and females.

**Table 1:** Data collection techniques and socio-demographic characteristics of participants

Data collection technique	Type of participants	Age group (years)	Sex		Total
			Male	Female	
IDI	CDD	25–52	6	4	10
	CHN	24–48	1	6	7
	District disease control officers	26–45	1	1	2
FGD	Community members	18–30	2 groups	2 groups	12 groups (6 participants in each group, thus, 72 participants)
		31–50	2 groups	2 groups	
		51 and above	2 groups	2 groups	

### Knowledge of NTD / MDA programmes

The respondents had knowledge of MDA activities in the communities. Respondents mentioned drug distributions and how the heights of community members are measured before drugs are given to them. This can be seen in the representative narrative below:

*“It’s very true, they usually bring drugs to share for all in the community (elderly and young). They use a stick in measuring”* (P2, Male, Agyambra, FGD).

*“They said with the drug when mosquito bites you, you will not get ‘Gyepim’ (Swollen feet) or ‘Otow’ (Swollen Scrotum). So, it makes them share the drugs step by step. They use a stick to measure. This helps us a lot”* (P1, Female, Asemkor, FGD).

As may be expected, the frontline healthcare providers have adequate knowledge about the MDA programmes for NTDs. They could describe the NTD programme as a collection of diseases that do not have enough funding for their activities. They could also talk about the processes involved in MDA implementation in the communities. Some of the processes described included that: drugs are brought from NTD programme office in the national capital (Accra) to the district health directorate, CDDs are invited to a workshop at the district level, where they participate in determining how to go about the distribution, determine the date it will start and assign supervisors. They also mentioned that the CDDs take the heights of participants and then use the treatment chart as a guide to give the appropriate number of tablets and then record this information in the treatment booklet. These views are represented in the following narratives:

*“I know everything about the MDA activities because I have been involved as a CDD for more than 12 years. […] as soon as our bosses at the district administration received the drugs, they call us to a workshop for refresher training and then plan on how to go about the distribution. At the workshop, we are assigned our supervisors. They also tell us how much will be given to us as daily allowance”* (Male CDD, Male, Bonsukrom, IDI)

*“I know that the NTD programme is in charge of the neglected tropical diseases, which do not have enough money for their control activities […] they are neglected. As for the MDA, we have been organising for many years now and it is unfortunate that we could not have it in this year (2020) because of COVID-19)”* (Female Community Health Nurse, Asemasa/Asemkor, IDI).

### Knowledge of COVID-19

Participants were generally aware of Coronavirus disease and referred to it appropriately by using the medically defined names such as ‘coronavirus’ and COVID-19. They often referred to it as ‘the new disease’ as captured in the responses below:

*“We know the new disease […] The new Disease is COVID-19”* (P5, Female, Nyameyekrom, FGD).

*“As a health worker, we have all the information on the disease, it is call coronavirus or COVI-19, which started from China* (Female Physician Assistant, Fasin Nyamekrom, IDI).

### Transmission of COVID-19

It came to light that most of the community respondents could not appropriately describe the route of transmission. They typically based their responses on the current control measures being promoted in the media. Most people reported that it is transmitted through touch or sharing of common items as captured in the following narratives:

*“What I know is that if someone gets infected and he or she speaks without having a nose mask on, those close by can get infected too, […]as to how the germs get into the person, I am not sure of it”* (P1, Male, Asemkor, FGD).

*“It is transmitted through the sharing of the same item. For example, with this plastic chair I am sitting on, if I am infected with the disease and another person comes to touch the plastic chair, that person can contract the disease”* (P2, Female, Mpatase, FGD).

The frontline healthcare workers on the other hand reported more correct routes of transmission of COVID-19 infection. They posited that the virus is transmitted through the nose, mouth, and eyes. This position is clearly captured in the representative narrative below.

*“The coronavirus is transmitted through the nose, mouth and the eyes. […] we know that COVID-19 is transmitted when droplets from an infected person* get into another person […] (Female Physician Assistant, Fasin Nyamekrom, IDI).

### Symptoms of COVID-19

Various symptoms were reported by community members and among them were; headache, cough, dryness in the throat, fever and painful lungs. These signs and symptoms are represented in the narratives below:

*“They say some of the symptoms are headache and coughs.”* (P3, Female, Bonsukrom, FGD)

*“Some of the signs are, the person will begin to cough, after a while the person will experience dryness in the throat and pains in the lungs. The person will also experience some feverishness”* (P1, Female, Mpatase, FGD)

Frontline healthcare workers also had vivid descriptions of the symptoms of the novel COVID-19 disease. Some of the reported symptoms were coughing, fever, breathing difficulty, dry throat, headache, bodily pains, and lung infections. These are captured in a representative response below:

*“As health workers, we know the various signs and symptoms associated with the COVID-19 disease. It usually starts like any febrile illness, presenting symptoms like, coughing, fever, bodily pains […]”* (Male District Disease Control officer, IDI).

### Prevention of COVID-19

Participants appropriately reported the current preventive measures being promoted in the media, which is an indication that information on personal hygiene and other protection protocols is reaching the people. These included the use of hand sanitizer, washing of hands, and sneezing appropriately as aptly represented in the following narratives:

*“We should use soap and water to regularly wash our hands. We should use hand sanitizers to clean our hands, and always keep a clean environment”* (P4, Female, Agyambra, FGD)

*“If you want to sneeze, you have to sneeze into your folded elbow* (showing the action) *so that the droplets do not come out to infect other people”* (P4, Male, Asemkor, FGD)

*“COVID-19, I believe can be prevented if all of us will follow the prevention protocols, especially washing our hands with soap under running water frequently, avoiding crowded places like churches, funerals, drinking spots. […] we must also wear our nose masks and/or face shields, use hand sanitizer in the absence of water, and keep physical distance when dealing with other people […] sneezing into handkerchiefs, tissue papers or your folded elbow”* (Female Physician Assistant, Fasin Nyamekrom, IDI).

### Effect of COVID-19 on daily lives/activities among community members

Participants expressed worries about how COVID-19 has negatively affected their daily lives and activities. Some of the ways by which the pandemic affected the daily activities of respondent were: general slowdown of business, especially among traders and store operators, inability to work due to restrictions in movement, fear of contracting the infection, and the fact that children cannot attend school as they used to. Below are some of the representative responses:

*“Business has slowed down due to this disease. People are not buying things like they used to”* (P2, Female, Achonwa, FGD).

*“I am not able to go for work as I used to. I am always at home now due to restrictions on movements and the fear of contracting the disease.”* (P4, Male, Adjumako, FGD).

*“First our children were going to school but now they are all home”* (P1, Female, Nyameyekrom, FGD).

### Effect of COVID-19 on NTD/MDA programme and service delivery

Generally, when asked about the effects of the COVID-19 pandemic on NTD programmes, the majority of CDDs stated that there is a negative effect on NTD programmes. They mentioned that the yearly MDA programme has been postponed due to the pandemic. Should the programme make the drugs available for distribution, some said they would be afraid to distribute drugs because one cannot tell who is infected. The following are some observations from respondents.

*“Okay, the programme is run yearly but look at the month we are in (September), because of COVID-19 the exercise could not come on”* (Male CDD, Adjumako/Mpatase, IDI).

*“Okay, first and foremost, with the presence of COVID-19, when delivering drugs, there is fear because you cannot determine if the receiver has it or not and therefore, you will be afraid to go round to distribute the drug. This will affect the work in a way”* (Male CDD, Bonsukrom, IDI).

*“We have heard that this COVID-19 is through contact or even a droplet from someone. So, it is difficult for you to go to someone and distribute the drug, because both the distributor and the receiver are afraid of each other”* (Male CDD, Nyamekrom, IDI).

Health workers in the facilities also expressed concerns about the effect of COVID-19 on NTD Programmes and service delivery. Just like the CDDs, they pointed out that the pandemic has affected the arrival and distribution of MDA drugs as presented in the following narratives:

*“The lockdown that we experienced at the earlier stage of the COVID-19 disease, has hindered the arrival of the drugs for distribution. This is the adverse effect of COVID-19 on the programme”* (Female Community Health Nurse, Asemasa/Asemkor, IDI).

*“Even the clinicians as well as the people they are going to administer those medications to, they are scared. They have some sort of fear in them that, “Okay, I do not know the home I am going to, I do not know who I am meeting, the kind of people he/she has been involved with, the past week, two or ever since of the outbreak of the pandemic”* (Female Physician Assistant, Fasin Nyamekrom, IDI).

The district health directorate also shared how the NTD programme and service delivery were affected. They maintained that COVID-19 was responsible for non-implementation of the yearly MDA. Also, people were hiding suspected signs and symptoms of COVID-19 from care providers. These positions were captured in the following narrative:

*“[…] basically, we started the year anticipating that we will have MDA activity; thus, mass drug administration programme on lymphatic filariasis, but along the line because of COVID-19 restrictions and the fact that we need to ensure that we go by the national COVID-19 protocols coupled with uncertainties in the air it could not come on. Basically, we were not able to do our scheduled mass drug administration for the year”* (Male District Disease Control Officer, IDI).

*“People who come to the facility with cough are hiding it from us […]. You see the person coughing and you ask “are you coughing? They would say no, only for them to go hiding in the then you hear ‘kuhu kuhu’(coughs). So, people are trying to hide some of the perceived signs and symptoms of COVID-19 from us (care providers) because they do not want to be tested for COVID-19”* (Male District Disease Control Officer, IDI).

Community volunteers expressed worry about the changes that have occurred at the health facilities since the advent of the COVID-19 pandemic. They were worried that patients were no longer receiving the kinds of services they used to receive from the care providers and that care providers were afraid to relate well to patients. The practice of social distancing has also affected operations at health facilities. These positions were represented in the following narratives:

*“The disease has made our attendance at hospitals very difficult, because the way and manner the nurses use to care for us has changed since the arrival of COVID-19”* (P2, Female, Agyambra, FGD).

*“Care providers don’t want to come closer to the patients and I think the social distancing protocol that is the reason why the doctors and nurses are behaving as such. The doctors are also afraid that maybe the patients have the disease (COVID-19) and if they don’t take the necessary precautions, they may get the infection”* (P3, Male, Agyambra, FGD).

*“There have been changes at the hospital. The bench that accommodates 6 persons previously, now can only accommodate 4 persons, making it difficult for all the patients to sit down comfortably. If you want to cough you have to go somewhere else. You can’t sit on the bench and cough. If you are tested and the result is positive you will be admitted and taken care of at the hospital”* (P1, Female, Bonsukrom, FGD).

### Effects of COVID-19 on attendance to health facility

All categories of respondents agreed that the COVID-19 pandemic has negatively affected clinic attendance. Health workers reported that they were getting worried because people with illnesses that they usually bring to the clinic were staying away from health facilities because of the fear of being diagnosed with COVID-19. This position was very aptly captured in the following representative narrative:

*“With the presence of COVID-19, it is really worrying, because people with illnesses that they may usually take to the clinic are now afraid to go to the clinic because they are afraid that they may be tested for COVID-19. They are afraid to be diagnosed to be having COVID-19, which may call for hospital admission and isolation. With this, it is difficult for people to seek healthcare. They prefer to stay with the sickness than being diagnosed of having COVID-19. I have seen someone who was sick and from my observation, I think it was malaria, but because of COVID-19 the person refused to attend clinic. […] when you are sick with Malaria and you seek healthcare with your high temperature, you can be detained for some time because of the high temperature. With situation like this it is difficult to seek treatment from the health facility”* (Male CDD, Bonsukrom, IDI).

Key informants at the district health directorate confirmed the reduction in clinic attendance at health facilities. They emphasized that the COVID-19 pandemic comes with some levels of stigma against those affected (both infected and their family members), so people did not want go to the clinic and get tested for COVID-19. Respondents also explained that some infections like COVID-19 could be contracted from the hospital/clinic, so, people want to avoid going to the clinic as a way of avoiding COVID-19. These positions are represented in the following narratives:

*“Even I myself, at a point in time when I had malaria, I felt like treating myself rather than going to the hospital […] for the fear of stigma associated with COVID-19”* (Male District Health Information Officer, IDI).

*“So, to a large extent, some people were not coming to the hospitals at all, because the hospital was even perceived to be a place where you can get nosocomial infections; that is Hospital Acquired Infections. So, we had a drastic dropped in clinic/hospital attendance”* (Male, District Disease Control officer, IDI).

### Ill-health experiences of Community members during COVID-19

Respondents shared their experiences of having fallen sick during this era of COVID-19. While some who suspected that they may be having the infection visited the hospital for testing, others did not go to the hospital, even though they were not well and needed clinical attention. These positions are well represented in the narratives below:

*“In March 2020, I felt ill, it coincided with the coming of coronavirus into Ghana, so I thought it was the virus and visited the hospital but after series of examinations. I was told it wasn’t COVID-19”* (P1, Male, Agyambra, FGD).

*“I felt sick around March 2020, but I didn’t have the courage to go to the hospital for testing but instead, I did self-medication […] I didn’t go to the hospital because I believed that when I go, they will test me and say I am infected with COVID-19* (P5, Male, Asemkor, FGD).

### Achieving compliance and confidence in MDA during COVID-19

Various views were expressed by respondents on how to increase compliance and confidence of community members in MDA in this period of COVID-19. Respondents believed that there is a need for more awareness creation or community education to help people understand that MDA could be implemented during the COVID-19 pandemic without problem. They also reported the need to follow the COVID-19 protocols, especially social distancing and wearing of nose masks. These positions were represented in the narratives below:

*“The CDDs have responsibility to educate the community to know that, though COVID-19 is there, Elephantiasis (LF) also exist. So, we should all come together and go by the protocols such as; wearing our nose masks, use sanitizer, wash our hands frequently, and that when we do that, we can continue with the MDA programme”* (Male CDD, Nyamekrom, IDI).

*“I think we can still go ahead with drug administration but ensuring that we follow the protocols that includes wearing of nose mask, ensuring that there is physical distancing. I think we can even take advantage of this to give more education and assurance to the community members that COVID-19 of course has come to stay but then when we follow the protocols, we can be able to bring it under control”* (Male District Disease Control officer, IDI).

### Physical distancing

Health workers, including CDDs, were concerned about physical distancing as a COVID-19 protocol. They find it very challenging because by the nature of their work, they have to get closer to their clients during MDA. Some of them asked rhetorically, “How can you give drugs to people without getting close to them?” Others were concerned about how to take the height of people to determine the number of tablets (drugs) to give them. These views were expressed in the following narratives:

*“As for social/physical distancing, it will be a challenge during MDA. Looking at how we share the drug, we take the height of the person, so if you do not get close to the person, how do you take the height? The social distancing […], we can find a way to practice it, but it will make the work a little more difficult”* (Male CDD, Adjumako/Mpatase, IDI).

*“The protocols have been laid down that is ensuring physical distancing. When you distance yourself, […] they will say you are behaving someway or indirectly you want to say the person has COVID-19 disease”* (Female Community Health Nurse, Agyambra, IDI).

### Usage of personal protective equipment

With regards to the use of PPE during MDA, respondents were divided on its potential effects on their work. While some did not see it as posing a challenge to MDA activities, others think it will create some challenges. Those who think that it will not pose any challenge believed that both the community members and CDDs must wear, at minimum, nose masks to prevent stigmatization. Those who said it will present some challenges reported that, wearing PPE, even the nose mask, will cause community members to challenge their sense of judgement; if they know it is not safe to implement MDA, why are they doing it? These positions were expressed in the following representative narratives:

*“Not adhering rather will cause them to ask us some questions like “we know we are in COVID-19 season and so why are you not adhering to the protocols?”* (Male CDD, Agyambra, IDI).

*“I think that, right now due to numerous announcement and advertisement concerning COVID-19, people have come to accept that this is the norm of the day […] but it will be good if the community members are also wearing it (face mask), so that when they see you in face mask, they will not think that you are stigmatizing them because of a condition or something”* (Female Physician Assistant, Fasin Nyamekrom, IDI).

*“They (community members) would not be comfortable seeing us in PPE, especially when they are not wearing some. They may be afraid of us. We are mostly in contentions with them on these COVID-19 protocols, as most of them believe that there is no COVID-19 in their communities”* (Female Community Health Nurse, Asemasa/Asemkor, IDI).

### Community response to MDA programmes

With regards to how community members will respond to MDA activities during this time, some respondents said that because community members are already aware of the programme, they may not respond differently. Others were of the view that some community members may link the MDA activities to COVID-19, which will make them hesitant to participate. These views are captured in the following narratives:

*“We already distribute the medicines to them, and they have been taking the medicines even before COVID-19. So, we will let them know that the medicines are not COVID-19 drugs but what they have already been taking. If we talk to them, they will understand”* (Male CDD, Asemkor, IDI).

*“I think rather that, the materials we need to protect ourselves, must also be made available to them, so that when they see us, they will know we are part of them and we are going to interact with them concerning the drug distribution”* (Female CDD, Achonwa, IDI).

### Hindrance to MDA participation

While community members believe there will be no hindrance to participation in the MDA, health workers think that there will be barriers to MDA implementation if appropriate measures are not put in place. Some of the recommended appropriate measures reported included community education and awareness, as well as observance of COVID-19 protocols. These positions were expressed in the following representative narratives:

*“We are not going to use Coronavirus as an excuse to stop taking the drug. The drugs help me with my health issues, so if you don’t continue this programme it is going to worry me”* (P3, Male, Bonsukrom, FGD).

*“Coronavirus is different from elephantiasis; hence we have to take elephantiasis drug in order to prevent it and avoid any complications”* (P1, Female, Adjumako, FGD).

*“The challenge will be that we will have to explain it well enough to them and answer some questions they may ask because of COVID-19. We the CDDs have to expect that and be prepared”* (Male CDD, Agyambra, IDI).

### Effective communication

For many respondents, effective and appropriate communication to create awareness and educate people was the best way to get community members to actively participate in MDA activities. They are of the view that any awareness creation in communities must involve the chiefs and elders as well as local political leaders in communities. This position is succinctly expressed in the following representative narratives:

*“First, I will suggest that we do an announcement and educate them on the sickness (COVID-19), as it has come to stay with us. What we need to do is to stay safe and then talk about the elephantiasis drug distribution and remind them that the drug distribution as is already known to them is to help us to eliminate elephantiasis”* (Male CDD, Bonsukrom, IDI).

*“In order for people to participate, we have to involve those in authorities like the chiefs or the assemblymen. When they speak, the community members will listen”* (P5, Female, Nyameyekrom, FGD).

*“Showing a video on the drug for them to watch will be useful. Most of them will come and watch it. When they watch and realized what the drug and disease is about, knowing the effect of not taking the drugs, knowing it is mosquitoes that cause it and mosquitoes are rampant in the communities then they will take the drug. If we do this it will help very much”* (Male CDD, Adjumako/Mpatase, IDI).

When health workers were asked about their roles in social mobilization to get community members to participate in the MDA activities, they said, talking to the people about the usefulness of the drugs through community elders, religious groups and other community durbars. These views are represented in the following narratives:

*“Our duty is to properly talk to them (community members) about the usefulness of the drugs. If we do that, they will take the drug”* (Female CDD, Agyambra, IDI).

*“The CDD can go from church to church to give education on the drugs and its usefulness. Aside the churches, they can go to the local radio stations as well to give talks, when they (CDDs) are supported by providing their needs and logistics”* (Male CDD, Nyamekrom, IDI).

*“My role will be ensuring that we start social mobilization ahead of time. If we are to do the MDA in a month time, we should start social mobilization ahead of time so that the community will be able to buy into the programme”* (Male District Disease Control officer, IDI).

### MDA implementation amidst COVID-19

Respondents were divided on implementing MDA amidst COVID-19. While some believed that MDA activities could be carried out without any problem provided the protocols are followed, especially wearing of nose masks and keeping physical distance, others think that it will be difficult to implement MDA activities amidst the COVID-19 pandemic. These positions were captured in the following narratives:

*“Oh yeah. It should continue. As I earlier indicated, though COVID-19 is currently available it doesn’t mean elephantiasis is not there. COVID-19 is just one of the conditions that may affect us”* (Male CDD, Nyamekrom, IDI).

*“We have a period for the distribution of the medicines. So, if the time is up, they should bring the medicines for distribution and we will do it by following the COVID-19 protocols”* (Male CDD, Asemkor, IDI).

*“I will suggest we hold on with the distribution. Somewhere next year, God willing, if the spread is contained. Now, although COVID-19 cases are going down, it is not completely gone. If we distribute the drug now, some will take it and others will not because of the fear of COVID-19”* (Male CDD, Bonsukrom, IDI).

## Discussions

In preparation for MDA, the WHO issued recommendations for safely restarting MDA for NTDs in settings with heightened risk of COVID-19 community transmission [22]. The recommendations aptly identify most of the adaptations necessary for conducting MDA during COVID-19. Furthermore, the recommendations also suggested the need for field teams in MDA areas to be consulted prior to restart to grasp the operational realities in field settings. The need to consult the community is what this paper aimed to address; beyond the planning, training, logistics and funding requirements for MDA which by themselves will represent significant challenges to programmes in addressing the multi-faceted challenges posed by COVID-19.

The perceptions from the CDDs, CHNs, disease control officers, and community members underscore the need to strengthen education on MDA as they saw it as a critical element to MDA during COVID-19. Participants expressed worry about the negative impact of COVID-19 on their daily lives and NTD programme activities, however, their major concern surrounded service delivery at the community and health facility levels. The fear of contracting COVID-19 and the resulting stigmatization represent major challenges to health service provision and must be addressed, to safely restart MDA activities and achieve effective coverage. Stigmatization due to COVID-19 [23, 24] has been reported as a major hurdle to an effective pandemic response. This is compounded by the fact that NTDs already carry a heavy burden of social stigma [25].

Respondents also expressed their views on how to increase compliance and confidence of community members for MDA during this period. The health workers, including CDDs, were concerned with physical distancing as required COVID-19 protocol. They found it very challenging because, by the nature of their work, they may have to get closer to their clients than guidelines allow. Respondents were divided on the effect of wearing PPE during MDA. For many respondents, effective and appropriate communication to create awareness and educate people on the need for MDA is the best way to get community members to actively participate in MDA activities. They believed that continuing with MDA and NTD programmes despite COVID-19 will not be a problem provided the protocols are followed.

In Ghana, and elsewhere, lymphatic filariasis MDA activities are already burdened with barriers to attaining optimal treatment coverage [26–29], facilitated by inaccurate reported data [30–32] and socio-cultural and health system challenges [33]. As such, more effort will be required to address the MDA challenges during the COVID-19 era. Innovative approaches to addressing the COVID-19 challenges to NTD programmes have been proposed: strengthening health systems, facilitating integration between diseases and cross-sector collaboration, improving and optimizing programme interventions delivery [34, 35] and these cannot be overemphasised. Successful implementation of such strategies across all NTD communities and countries will be key to attaining the NTD 2030 elimination targets post-COVID-19.

In conclusion, we found that COVID-19 and its preventive protocols have led to some negative perceptions about restarting MDA activities among our study participants. These negative perceptions among community members, CDDs, and health workers must be addressed to enhance participation in MDA activities, else the impacts of COVID-19 may reverse the significant gains made in reducing the NTD, especially LF, burden in the last 20 years.

## Supplementary Information

**Additional file 1. Table S1.** Thematic categories with selected quotations from the narrative responses..

## Data Availability

Qualitative data generated and analysed during this study are included in this published article. Raw data is not available and will not be shared, as this would compromise the protection of participants’ identity, however, some data could be made available upon a reasonable request from the corresponding author, cahorlu@noguchi.ug.edu.gh.

## Abbreviations

CDD: Community-based Drug Distributors
CHN: Community Health Nurse
COVID-19: Coronavirus Disease
FGD: Focus Group Discussion
FWA: Federal Wide Assurance
GPELF: Global Programme to Eliminate Lymphatic Filariasis
LF: Lymphatic Filariasis
MDA: Mass Drug Administration
NMIMR-IRB: Noguchi Memorial Institute for Medical Research Institutional Review Board
IDI: In-depth Interview
NTD: Neglected Tropical Disease
PPE: Personal Protective Equipment
