# Supplementary material for: COVID-19 related perception among some community members and frontline healthcare providers for NTD control in Ghana
Source: BMC Infect Dis. 2022 Jan 30;22:106. doi: 10.1186/s12879-022-07084-0 (PMC8801039; doi:10.1186/s12879-022-07084-0)
Supplement: Supplementary file 1 — Additional file 1. Table S1. Thematic categories with selected quotations from the narrative responses.. [file 12879_2022_7084_MOESM1_ESM.docx]

Table S1: Thematic categories with selected quotations from the narrative responses

| Thematic Codes | Health Workers | CDDs | Community |
| --- | --- | --- | --- |
| **Knowledge of NTD / MDA programmes** | *“I know that the NTD programme is in-charge of the neglected tropical diseases, which do not have enough money for their control activities […] they are neglected. As for the MDA, we have been organising for many years now and it is unfortunate that we could not have it in this year (2020) because of COVID-19)”* (Female Community Health Nurse, Asemasa/Asemkor, IDI). | *“I know everything about the MDA activities because I have been involved as a CDD for more than 12 years. […] as soon as our bosses at the district administration received the drugs, they call us to a workshop for refresher training and then plan on how to go about the distribution. At the workshop, we are assigned our supervisors. They also tell us how much will be given to us as daily allowance”* (Male CDD, Male, Bonsukrom, IDI). | *“[…] they said with the drug, when mosquito bites you, you will not get ‘Gyepim’ (Swollen feet) or ‘Otow’ (Swollen Scrotum). So, it makes them share the drugs step by step. They use a stick to measure. This helps us a lot”* (P1, Female, Asemkor, FGD). |
| **Knowledge of COVID-19** | *“As a health worker, we have all the information on the disease, it is call coronavirus or COVI-19, which started from China* *(**Female Physician Assistant, Fasin Nyamekrom, IDI).* | *“As for covid, if you are in Ghana and you don’t know about it then there is something wrong with you, […] I have been educating people to wear their nose masks* (Female CDD, Asemkor, IDI*).* | *“We know the new disease […] The new Disease is COVID-19” (P5, Female, Nyameyekrom, FGD).* |
| **Transmission of COVID-19** | *“The coronavirus is transmitted through the nose, mouth and the eyes. […] we know that COVID-19 is transmitted when droplets from an infected person get into another person […] (Female Physician Assistant, Nyamekrom, IDI).* | *Once we are told to cover our mouth and nose, it means that we can get it when someone cough into our faces, or when you touch the germs and with your hands and use it to touch your mouth or pick your nose, you can get it* (Male CDD, Bonsukrom, IDI). | *“What I know is that if someone gets infected and he or she speaks without having a nose mask on, those close by can get infected too, […]as to how the germs get into the person, I am not sure of it”* (P1, Male, Asemkor, FGD). |
| **Symptoms of COVID-19** | *“As health workers, we know the various signs and symptoms associated with the COVID-19 disease. It usually starts like any febrile illness, presenting symptoms like, coughing, fever, bodily pains […]”* (Male District Disease Control officer, IDI). | *“From the education on the radio and TV, I got to know that when you have the disease, you show the following signs, fever, coughing, bodily pains and difficulty in breathing […]”* (Female CDD, Asemkor, IDI). | *“Some of the signs are the person will begin to cough, after a while the person will experience dryness in the throat and pains in the lungs. The person will also experience some feverishness”* (P1, Female, Mpatase, FGD). |
| **Prevention of COVID-19** | “*COVID-19, I believe can be prevented if all of us will follow the prevention protocols […] washing our hands with soap under running […], avoiding crowded places like churches, funerals etc. We must wear our nose masks […], use hand sanitizer […], keep physical distance when dealing with other people […] sneezing into handkerchiefs, tissue papers or your folded elbow*” (Female Physician Assistant, Fasin Nyamekrom, IDI). | “I believe that the most important way to prevent COVID-19 is to wear your nose mask always when you are not at home. Use hand sanitizer and wash your hands with soap frequently (Male CDD, Mpatase, IDI). | *“We should use soap and water to regularly wash our hands. We should use hand sanitizers to clean our hands, and always keep a clean environment”* (P4, Female, Agyambra, FGD).  *“If you want to sneeze, you have to sneeze into your folded elbow* (showing the action) *so that the droplets do not come out to infect other people”* (P4, Male, Asemkor, FGD). |
| **Effect of COVID-19 on daily lives/activities of community members** | “*At the very early stage of the pandemic, it has very devastating effect of the lives if the people. Nothing was working, people cannot travel to big towns to do business, in fact, nothing was working but it is getting better now”* ((Male, District Disease Control officer, IDI).  *“In most communities, live has virtually come to a halt, nothing seems to be working anymore. Even people have stop coming to the clinic and only shown up as emergency cases. The situation is bad”* (Female Community Health Nurse, Asemasa/Asemkor, IDI). | *“I can say that I have not seen anything that has affected the life of the pwople like this evil pandemic. It has affected every aspect of our lives, we cannot even go to the farm, even if you go, you cannot find buyers for your farm produce. It was very bad situations and people are really suffering, you cannot even travel, children cannot go to school all due to COVID-19 […]”* (Male CDD Bonsukrom). | *“Business has slowed down due to this disease. People are not buying things like they used to”* (P2, Female, Achonwa, FGD).  *“I am not able to go to work as I used to. I am always at home now due to restrictions on movements and the fear of contracting the disease.”* (P4, Male, Adjumako, FGD).  *“First our children were going to school but now they are all home”* (P1, Female, Nyameyekrom, FGD). |
| **Effect of COVID-19 on NTD / MDA programme and service delivery** | *“The lockdown that we experienced at the earlier stage of the COVID-19 disease, has hindered the arrival of the drugs for distribution. This is the adverse effect of COVID-19 on the programme”* (Female Community Health Nurse, Asemasa/Asemkor, IDI).  *“[…] basically, we started the year 2020, anticipating that we will have MDA activity […] for lymphatic filariasis, but along the line because of COVID-19 restrictions and the fact that we need to ensure that we go by the national COVID-19 protocols coupled with uncertainties […] we were not able to do our scheduled MDA for the year 2020”* (Male District Disease Control Officer, IDI). | *“Okay, the programme is run yearly but look at the month we are in (September), because of COVID-19 the exercise could not come on”* (Male CDD, Adjumako / Mpatase, IDI).  *“Okay, first and foremost, with the presence of COVID-19, when delivering drugs, there is fear because you cannot determine if the receiver has it or not and therefore, you will be afraid to go round to distribute the drug. This will affect the work in a way”* (Male CDD, Bonsukrom, IDI). | *“The disease has made our attendance at hospitals very difficult, because the way and manner the nurses use to care for us has changed since the arrival of COVID-19”* (P2, Female, Discove, FGD).  *“Care providers don’t want to come closer to the patients and I think the social distancing protocol is the reason why the doctors and nurses are behaving as such. The doctors are also afraid that maybe the patients have the disease (COVID-19) and if they don’t take the necessary precautions, they may get the infection”* (P3, Male, Agyambra, FGD). |
| **Effects of COVID-19 on attendance to health facility** | *“Even I myself, at a point in time when I had malaria, I felt like treating myself rather than going to the hospital […] for the fear of stigma associated with COVID-19”* (Male District Health Information Officer, IDI).  *“So, to a large extent, some people were not coming to the hospitals at all, because the hospital was even perceived to be a place where you can get nosocomial infections; that is Hospital Acquired Infections. So, we had a drastic dropped in clinic/hospital attendance”* (Male, District Disease Control officer, IDI). | *“With the presence of COVID-19, it is really worrying, because people with illnesses that they may usually take to the clinic are now afraid to go to the clinic because they are afraid that they may be tested for COVID-19. They are afraid to be diagnosed to be having COVID-19, which may call for hospital admission and isolation. With this, it is difficult for people to seek healthcare. They prefer to stay with the sickness than being diagnosed of having COVID-19. I have seen someone who was sick and from my observation, I think it was malaria, but because of COVID-19 the person refused to attend clinic. […] when you are sick with Malaria and you seek healthcare with your high temperature, you can be detained for some time because of the high temperature. With situation like this it is difficult to seek treatment from the health facility”* (Male CDD, Bonsukrom, IDI). | *“Most of us are Afraid to go to the hospital because, as soon as you cough or have fever, they will suspect COVID-19 and then ask you not to go into public places until your test result is out […] sometimes they even send you to the hospital for admission till the result comes. You most of us will not go until the situation becomes very severe and at that point you cannot refused […]. I now buy drugs from the drug store, even for my children […]”* (P 3, Female, Mpatano, FGD).  *“People are not going to the clinic at all […] if you pass by the clinic, the place is empty most of the time. People are afraid of the COVID-19 label because once you are suspected and your sample is taken, everybody in the community will hear that you have COVOI-19, even if your result is negative”* (P2, Male, Agyambra, FGD). |
| **Ill-health experiences of Community members during COVID-19** | *“When you go through the community, you can see that some people are not well, but they won’t come to the clinic and when you ask them, they say, they have bought medicines from the drug vendors, all because of COVID, people are increasingly resorting to self-medications and the use traditional remedies”* Nurse, Asemasa/Asemkor, IDI). | COVID-19 has changes everyone’s experiences of ill-health. Now people are going back to traditional medicines, taking all kinds of herbal teas […] for some people even when you can tell that they are sick, they will tell you that they were just tired” (Male CDD, Mpatase, IDI). | *“I felt sick around March 2020, but I didn’t have the courage to go to the hospital for testing but instead, I did self-medication […] I didn’t go to the hospital because I believed that when I go, they will test me and say I am infected with COVID-19* (P5, Male, Asemkor, FGD). |
| **Achieving compliance and confidence in MDA during COVID-19** | *“I think we can still go ahead with drug administration but ensuring that we follow the protocols that includes wearing of nose mask, ensuring that there is physical distancing. I think we can even take advantage of this to give more education and assurance to the community members that COVID-19 of course has come to stay but then when we follow the protocols, we can be able to bring it under control”* (Male District Disease Control officer, IDI). | *“The CDDs have responsibility to educate the community to know that, though COVID-19 is there, Elephantiasis (LF) also exist. So, we should all come together and go by the protocols such as wearing our nose masks, use sanitizer, wash our hands frequently, and that when we do that, we can continue with the MDA programme”* (Male CDD, Nyamekrom, IDI). | “*I think that when they give everybody in the community face mask and sanitizer, we shall participate in taking of the drugs”* (P4, Female, Bonsukrom, FGD).  *“The timing of the MDA activity and how it is communicated to us in the community and the assurance of protecting us from COVID-19, will help a lot to make people to take the dug’* (P5, Mal, Asemkor, FGD). |
| **Physical distancing during COVOD-1** | *“The protocols have been laid down that is ensuring physical distancing. When you distance yourself, […] they will say you are behaving someway or indirectly you want to say the person has COVID-19 disease”* (Female Community Health Nurse, Agyambra, IDI). | *“As for social/physical distancing, it will be a challenge during MDA. Looking at how we share the drug, we take the height of the person, so if you do not get close to the person, how do you take the height? The social distancing […], we can find a way to practice it, but it will make the work a little more difficult”* (Male CDD, Mpatase, IDI). | “*It will be difficult to practice social distancing during drug distribution because I must collect the drug from the hand of the CDD, so how can I distance myself from him/her? I think the best is that we all wear face masks and then looking away when talking to each that, but that also in not culturally acceptable to us”* (P6, Male Adjumako, FGD). |
| **Usage of personal protective equipment** | *“I think that, right now due to numerous announcement and advertisement concerning COVID-19, people have come to accept that this is the norm of the day […] but it will be good if the community members are also wearing it (face mask), so that when they see you in face mask, they will not think that you are stigmatizing them because of a condition or something”* (Female Physician Assistant, Fasin Nyamekrom, IDI). | *“Not adhering rather will cause them to ask us some questions like “we know we are in COVID-19 season and so why are you not adhering to the protocols?”* (Male CDD, Agyambra, IDI). | “*We are not used to wearing it, so you see people covering only their mouth only, others also put it under their chin. […] You can also not blame people for not wearing it because it is not everybody who can afford it, it will be good if the Government supply it to all of us, just like you have given them to us before talking to us. As for sanitizer, only few people have them in this community”* (P3, Female Mpatase, FGD). |
| **Community response to MDA programme during COVID-19** | *“I think it may not be very good, if we do not invest time and resources to educate them on the importance of participating in MDA at this time of COVID-19. Some people may participate willingly but for others, more efforts may have to go into convincing them. They may hide behind the physical distancing protocol to stay away from the CDDs”* (Male, District Disease Control officer, IDI). | *“I think that, the materials we need to protect ourselves, must also be made available to community members, so that when they see us, they will know we are part of them and we are going to interact with them concerning the drug distribution”* (Female CDD, Achonwa, IDI). | *Me and my family, we shall take the drugs, but the CDD must be in fgace masks and give us some distance, when giving us the drugs”* (P5, Female, Agyambra, FGD).  *“I will take the drug only when all of us, CDD and myself, are wearing nose mask and I see the CDD sanitizing her hands before giving me the drug”* (P3, Male, Adjumako, FGD) |
| **COVID-19 as a hindrance to MDA participation** | *“As for COVID-19, it has affected many aspects of health delivery […] There was no MDA last year and this year (2021) we are only hearing rumours that it will come on. I am aware that some of the drugs are getting closer to expiring”* ((Female, District Disease Control officer, IDI). | *“The challenge will be that we will have to explain it well enough to them and answer some questions they may ask because of COVID-19. We the CDDs have to expect that and be prepared”* (Male CDD, Agyambra, IDI). | *“I know for sure that some people will not participate in the MDA because of COVID […], people who are coughing will not like anybody to suspect them of having covid, so they will stay away from the MDA”* (P2, Female, Achowa, FGD). |
| **Effective communication** | *“The CDD can go from church to church to give education on the drugs and its usefulness. Aside the churches, they can go to the local radio stations as well to give talks, when they (CDDs) are supported by providing their needs and logistics”* (Male CDD, Nyamekrom, IDI). | *“First, I will suggest that we do an announcement and educate them on the sickness (COVID-19), as it has come to stay with us. What we need to do is to stay safe and then talk about the elephantiasis drug distribution and remind them that the drug distribution, as is already known to them, is to help us to eliminate elephantiasis from the community”* (Male CDD, Bonsukrom, IDI). | I think that they (CDDS) should give us ample time to prepared for them […] They should come into the community to inform us two or more times before the distribution […] when they do that, that people will be prepared to participate” P3, Male Mpatano, FGD). |
| **MDA implementation amidst COVID-19** | *“I sincerely believe that it is not in the interest of the programme not to implement MDA this year since it was not done last year. We should just follow the protocols by wearing of nose mask and washing our hand frequently during MDA activity in communities”* (Female Nurse, Bonsukrom). | *“Oh yeah. It should continue. As I earlier indicated, though COVID-19 is currently available it doesn’t mean elephantiasis is not there. COVID-19 is just one of the conditions that may affect us”* (Male CDD, Nyamekrom, IDI).  *“I will suggest we hold on with the distribution till 2022, God willing, if the spread is contained […]. If we distribute the drug now, some will take it and others will not because of the fear of COVID-19”* (Male CDD, Bonsukrom, IDI). | *“Like I said earlier, the drug distribution (MDA) has helped to reduce the disease (LF) in this community […] I think it must be done but we (CDDs and community members) must wear nose masks during the distribution* (P2 Female, Bonsukrom, FGD).  *[…] I am not sure how it can be done with all the COVID-19 Protocols in place, but for the sake of the children, it should be done this year […]”* (P4, Male, Asemkor, FGD). |
